# A new species of ice cream cone worm in the Gulf of California (Annelida, Pectinariidae)

**DOI:** 10.3897/BDJ.10.e94772

**Published:** 2022-10-27

**Authors:** María Ana Ana Tovar-Hernández, Jesús Angel de León-González

**Affiliations:** 1 Universidad Autónoma de Nuevo León, Facultad de Ciencias Biológicas, Laboratorio de Biosistemática, San Nicolás de los Garza, Nuevo León, Mexico Universidad Autónoma de Nuevo León, Facultad de Ciencias Biológicas, Laboratorio de Biosistemática San Nicolás de los Garza, Nuevo León Mexico

**Keywords:** *Pectinaria*, Polychaeta, Terebellomorpha, Sea of Cortes, new species

## Abstract

**Background:**

*Pectinaria* Lamarck, 1818 is composed of 30 species, three of them were originally described from the west coast of the USA and Mexico: *P.californiensis* Hartman, 1941 (from Redondo Beach, California, USA), *P.newportensis* Hartman, 1941 (from Newport Bay, California, USA) and *P.hartmanae* Reish, 1968 (from Bahia de los Angeles, north-western Gulf of California, Mexico).

**New information:**

A new pectinariid polychaete, *Pectinariasantii* sp. n., is reported from the central-eastern Gulf of California, Mexico. *Pectinariasantii* sp. n. is clearly distinguished from its congeners by a combination of the following morphological features: segment 4 with a ventral crest with six horn-shaped anterior projections; three chaetigers with notopodia (S5, S6 and S7); 12 chaetigers with noto- and neuropodia (from S8–19); 8–10 golden opercular paleae per lobe; a pair of ear-shaped lobes at the base of the cephalic veil; an anterior row of chaetae on notopodia with a deep incision and a bifid process at the lateral end of the shaft; and 13–21 pairs of amber scaphal hooks with distal margin rounded and hooded. A full description, including variation and photographs in live and fixed specimens, is provided, as well as a key to the species of *Pectinaria* from the Tropical Eastern Pacific and Temperate Northern Pacific.

## Introduction

Pectinariids are polychaete worms that are easily recognised by their distinctive tubes which resemble the texture of an ice cream cone. They are made of cemented sand grains, small stones and shell fragments. These worms also have a characteristic set of golden paleae at the margin of the operculum. They use these structures for digging in soft sediments (Hutchings et al. 2020) which they inhabit with the cephalic region pointing downwards and posterior end orientated upwards to the surface (García-Garza and de León-González 2014).

The bodies of all members of the Pectinariidae are conical and short, with 26 segments in all species. This family comprises the genera *Amphictene* Savigny, 1822 (Savigny 1822), *Cistenides* Malmgren, 1866 (Malmgren 1866), *Lagis* Malmgren, 1866 (Malmgren 1866), *Pectinaria* Lamarck, 1818 (Lamarck 1818) and *Petta* Malmgren, 1866 (Malmgren 1866), with a total of more than 60 species (Hutchings et al. 2020).

*Pectinaria* is the most speciose genus, with 30 species (Zhang et al. 2022). The distinctive features of the genus *Pectinaria* are the presence of a cirrated cephalic veil, completely free from the opercular lobe, with numerous long cirri on the rim; dorsal and lateral opercular margin smooth; comb-like branchiae present on segments 3–⁠4; pectinate uncini have at least two longitudinal rows of evenly-sized teeth and a stout handle; and the scaphe is flattened dorsally, with anal flap and distinctly separated from the posterior segments (Nogueira et al. 2019, Zhang and Hutchings 2019, Hutchings et al. 2020, Zhang et al. 2022).

Until a few years ago, the most important diagnostic characters to identify species of *Pectinaria* were as follows: the number of cirri on the cephalic veil, the number of pairs of paleae, the morphology of the uncini and scaphal hooks, the presence of a postero-dorsal lobe on segment 2 and a mid-dorsal cirrus in the anal flap (Zhang and Qiu 2017). However, characters like the numbers of cirri on the cephalic veil, presence of a postero-dorsal lobe, the pairs of paleae and number of scaphal hooks are discrete measurements, with overlapping ranges amongst several species which makes comparison amongst species difficult. In recent publications, additional characters have been proposed to identify species, such as the presence of a pair of ear-shaped lobes which are adjacent to the dorsal base of the cephalic veil and ventral lappets on the lateral margin of segment 1 (Zhang and Hutchings 2019, Nogueira et al. 2019, Zhang et al. 2022).

A comprehensive table of diagnostic characters of all *Pectinaria* species recognised was provided by Zhang and Hutchings (2019). Pectinariids from Australia (Hutchings and Peart 2002, Wong and Hutchings 2015, Zhang and Hutchings 2019, Zhang et al. 2019), China, Korea and Japan (Sun and Qiu 2012, Nishi et al. 2014, Zhang et al. 2015, Choi et al. 2017, Zhang and Qiu 2017, Zhang et al. 2022), Brazil (Nogueira et al. 2019), Iceland (Papapar et al. 2020), Scandinavia and northern Europe (Holthe 1986a, Holthe 1986b) have the best documented pectinariid fauna.

From the west coast of the USA and Mexico, only three species of *Pectinaria* have been described: *P.californiensis* Hartman, 1941 (Hartman 1941) (from Redondo Beach, California, USA), *P.newportensis* Hartman, 1941 (Hartman 1941) (from Newport Bay, California, USA) and *P.hartmanae* Reish, 1968 (Reish 1968) (from Bahia de los Angeles, north-western Gulf of California, Mexico).

*Pectinariacaliforniensis* and *P.newportensis* have been reported in some ecological studies in the southern Gulf of California (Villalobos-Guerrero and Molina-Acevedo 2014 and references therein), but diagnoses, illustrations or taxonomic comments were not provided. Consequently, their comparison and identity cannot be determined. *Pectinariahartmanae* has not been recorded since it was described.

In this study, several specimens of *Pectinaria* were sampled in an estuary located in the central-eastern Gulf of California, but these worms have some features that do not fit with any of the currently known species in the genus. A full description including variation and photographs of both live and fixed specimens are provided.

## Materials and methods

**Figure 1.**
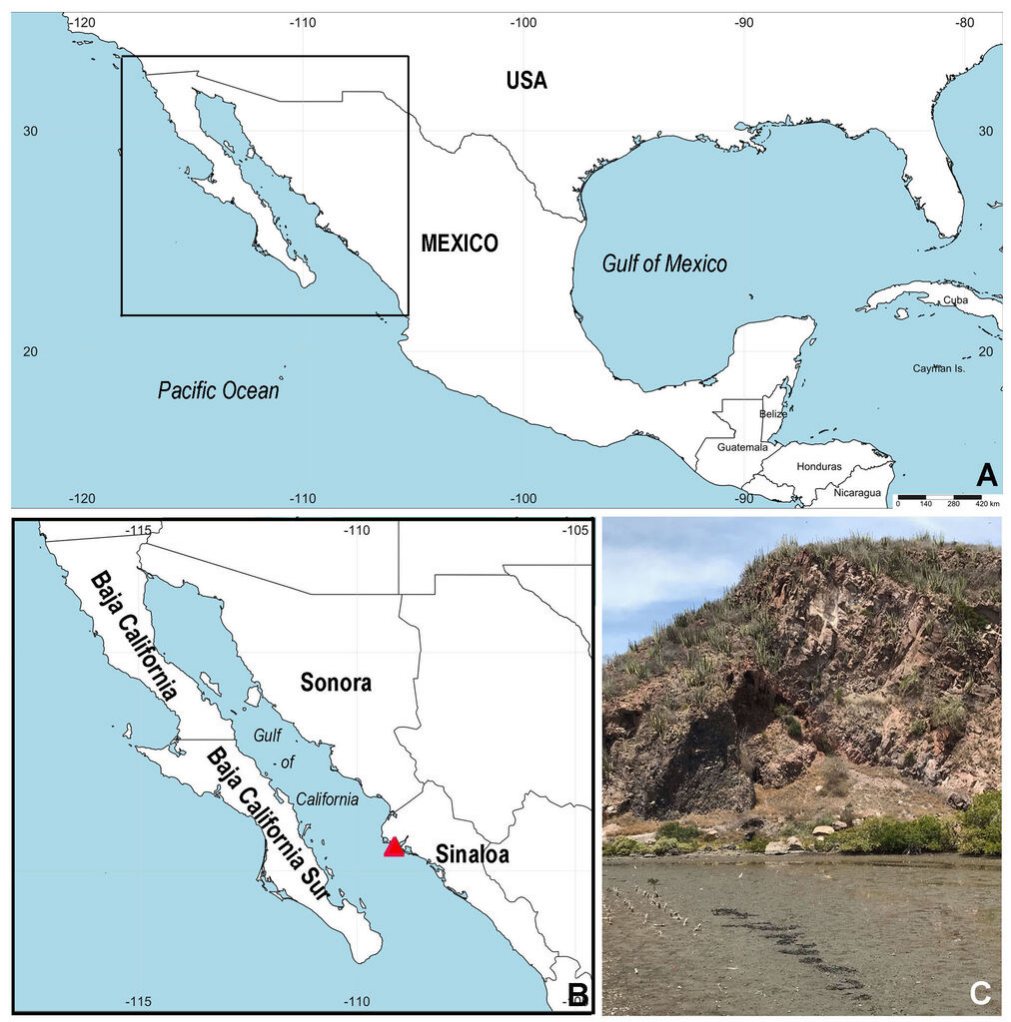
Sampling area. **A–C)** Location of sampling site, red triangle; **B** indicates type locality in El Mavíri.

Samples were collected by hand using a garden shovel during low tide at the Maviri Estuary, central-eastern Gulf of California (Fig. 1). Tubes were placed in a portable aquarium with air stones for three hours to document live colouration. Initial photographs were taken with an attached Nikon D610 digital camera. Then, worms were placed in the fridge at 4ºC for 30 minutes to relax and then fixed in 96% ethanol.

Specimens were studied using stereomicroscopes and representative types were photographed. Methyl Green dissolved in 75% ethanol was used to improve the contrast of features, measurements and analysis of the main morphological features. Under a stereomicroscope, the specimens were kept in the desired positions with a glass coverslip and photographed from a Petri dish with a black base. Notochaetae and neuropodia were removed from anterior and posterior regions of the body, mounted on slides with glycerine and examined using compound microscopes and photographed with an attached Canon EOS Rebel T7i digital camera. All photos were edited with Adobe Photoshop CS6 software. Line drawings of chaeta and scaphal hooks were done, based on images taken with the Canon EOS Rebel T7i.

Holotype and paratypes were measured post-fixation to record body length (from the anterior margin of the operculum to the last segment of scaphe), body width at chaetiger 3, number of cirri on cephalic veil, number of cephalic palea on right bundle, operculum width and number of scaphal hooks on the right bundle (Table 1). In the description section, these measurements are given for the holotype with the range for paratypes within parenthesis.

Nomenclature for morphological features follows Zhang and Hutchings (2019), Nogueira et al. (2019), Hutchings et al. (2020) and Zhang et al. (2022).

Two additional specimens from Boca del Camichín (Mexico, Nayarit: 21°44'38.0"N, 105°29'26.2"W), were provided by Patricia Salazar Silva (Instituto Tecnológico de Bahía Banderas) which agree with the new species here named.

Holotype and paratypes of *Pectinariahartmanae* Reish, 1968 (Reish 1968) were kindly examined by Dr. Karen Osborn (Smithsonian Institution, United States National Museum, accession numbers 38398, 383999) to determine the presence or absence of the ventral crest with horn-shaped anterior projections and compared with the new species.

Series of types were deposited in the following collections: Colección Poliquetológica from Universidad Autónoma de Nuevo León, México (UANL); Colección Regional de Invertebrados Marinos, Instituto de Ciencias del Mar y Limnología, Universidad Nacional Autónoma de México (ICML–EMU); Colección Nacional de Anélidos Poliquetos de México, Instituto de Ciencias del Mar y Limnología, Universidad Nacional Autónoma de México (CNAP–ICML, UNAM), GEOMARE, A. C, Mazatlán, México (GEOMARE), El Colegio de la Frontera Sur (ECOSUR) and Benthic Invertebrate Collection of SCRIPPS Institution of Oceanography (SCRIPPS).

## Taxon treatments

### Pectinaria santii

Tovar-Hernández & de León-González sp. n.

848DF57D-4C2D-526F-9BF7-3CF0BD1B53B7

13A59ACE-1FCB-441A-B9B4-D4EC6A898583

#### Materials

- **Type status:** Holotype. **Occurrence:** occurrenceRemarks: in mud, 37 ppt, 29ºC; recordNumber: TOP-S-20210408-2; recordedBy: Santiago Hernández and María Ana Tovar-Hernández; occurrenceID: 24FCB8F0-D835-5D16-A4AD-A66FE0994D20; **Taxon:** kingdom: Animalia; phylum: Annelida; class: Polychaeta; order: Terebellomorpha; family: Pectinariidae; genus: Pectinaria; specificEpithet: *santii*; taxonRank: species; taxonomicStatus: a; **Location:** higherGeography: North America, México, Gulf of California; continent: America; waterBody: Gulf of California; country: Mexico; countryCode: MX; stateProvince: Sinaloa; municipality: Ahome; locality: El Mavirí; verbatimDepth: 0.2 m; verbatimLatitude: 25º34’55’’N; verbatimLongitude: 109º6’53’’W; **Identification:** identifiedBy: María Ana Tovar-Hernández; **Event:** samplingEffort: 2 collectors, 2 hours sampling; eventDate: 10 am; year: 2021; month: April; day: 8; habitat: Estuary; fieldNumber: TOP-S-20210408-2; **Record Level:** institutionID: SCRIPPS; collectionID: SIO-BIC; collectionCode: SIO-BIC A13452
- **Type status:** Paratype. **Occurrence:** recordNumber: TOP-S-20210408-2; individualCount: 32; occurrenceID: 9C3A6C82-B251-5F36-BC43-BBA670CAF64C; **Taxon:** kingdom: Animalia; phylum: Annelida; class: Polychaeta; order: Terebellomorpha; family: Pectinariidae; genus: Pectinaria; specificEpithet: *santii*; taxonRank: species; taxonomicStatus: a; **Location:** higherGeography: North America, México, Gulf of California; continent: America; waterBody: Gulf of California; country: Mexico; countryCode: MX; stateProvince: Sinaloa; municipality: Ahome; locality: El Mavirí; verbatimDepth: 0.2 m; verbatimLatitude: 25º34’55’’N; verbatimLongitude: 109º6’53’’W; **Identification:** identifiedBy: María Ana Tovar-Hernández; **Event:** samplingEffort: 2 collectors, 2 hours sampling; year: 2021; month: April; day: 8; habitat: Estuary; fieldNumber: TOP-S-20210408-2; **Record Level:** institutionID: SCRIPPS; collectionID: SIO-BIC; collectionCode: SIO-BIC A13453
- **Type status:** Paratype. **Occurrence:** recordNumber: TOP-S-20210408-2; individualCount: 7; sex: 1 gravid female with asyncronous oocytes; occurrenceID: 59359B71-4B6B-5455-873E-23FB78B13DC3; **Taxon:** kingdom: Animalia; phylum: Annelida; class: Polychaeta; order: Terebellomorpha; family: Pectinariidae; genus: Pectinaria; specificEpithet: *santii*; taxonRank: species; taxonomicStatus: a; **Location:** higherGeography: North America, México, Gulf of California; continent: America; waterBody: Gulf of California; country: Mexico; countryCode: MX; stateProvince: Sinaloa; municipality: Ahome; locality: El Mavirí; verbatimDepth: 0.2 m; verbatimLatitude: 25º34’55’’N; verbatimLongitude: 109º6’53’’W; **Identification:** identifiedBy: María Ana Tovar-Hernández; **Event:** samplingEffort: 2 collectors, 2 hours sampling; year: 2021; month: April; day: 8; habitat: Estuary; fieldNumber: TOP-S-20210408-2; **Record Level:** institutionID: UANL; collectionID: UANL NL INV 002-05-09; collectionCode: UANL 8152
- **Type status:** Paratype. **Occurrence:** recordNumber: TOP-S-20210408-2; individualCount: 5; occurrenceID: 359902DE-2541-5F1A-B236-386771437CD7; **Taxon:** kingdom: Animalia; phylum: Annelida; class: Polychaeta; order: Terebellomorpha; family: Pectinariidae; genus: Pectinaria; specificEpithet: *santii*; taxonRank: species; taxonomicStatus: a; **Location:** higherGeography: North America, México, Gulf of California; continent: America; waterBody: Gulf of California; country: Mexico; countryCode: MX; stateProvince: Sinaloa; municipality: Ahome; locality: El Mavirí; verbatimDepth: 0.2 m; verbatimLatitude: 25º34’55’’N; verbatimLongitude: 109º6’53’’W; **Identification:** identifiedBy: María Ana Tovar-Hernández; **Event:** samplingEffort: 2 collectors, 2 hours sampling; year: 2021; month: April; day: 8; habitat: Estuary; fieldNumber: TOP-S-20210408-2; **Record Level:** institutionID: UNAM-ICML; collectionID: ICML-EMU; collectionCode: ICML-EMU-13290
- **Type status:** Paratype. **Occurrence:** recordNumber: TOP-S-20210408-2; individualCount: 5; sex: 1 gravid female with asyncronous oocytes; occurrenceID: 5E249DF2-F6AB-5175-BE70-8764BD28BF9A; **Taxon:** kingdom: Animalia; phylum: Annelida; class: Polychaeta; order: Terebellomorpha; family: Pectinariidae; genus: Pectinaria; specificEpithet: *santii*; taxonRank: species; taxonomicStatus: a; **Location:** higherGeography: North America, México, Gulf of California; continent: America; waterBody: Gulf of California; country: Mexico; countryCode: MX; stateProvince: Sinaloa; municipality: Ahome; locality: El Mavirí; verbatimDepth: 0.2 m; verbatimLatitude: 25º34’55’’N; verbatimLongitude: 109º6’53’’W; **Identification:** identifiedBy: María Ana Tovar-Hernández; **Event:** samplingEffort: 2 collectors, 2 hours sampling; year: 2021; month: April; day: 8; habitat: Estuary; fieldNumber: TOP-S-20210408-2; **Record Level:** institutionID: UNAM-ICML; collectionID: CNAP-ICML UNAM; collectionCode: POP-67-001
- **Type status:** Paratype. **Occurrence:** recordNumber: TOP-S-20210408-2; individualCount: 5; occurrenceID: E649117D-3138-51C1-B026-3D400AF45415; **Taxon:** kingdom: Animalia; phylum: Annelida; class: Polychaeta; order: Terebellomorpha; family: Pectinariidae; genus: Pectinaria; specificEpithet: *santii*; taxonRank: species; taxonomicStatus: a; **Location:** higherGeography: North America, México, Gulf of California; continent: America; waterBody: Gulf of California; country: Mexico; countryCode: MX; stateProvince: Sinaloa; municipality: Ahome; locality: El Mavirí; verbatimDepth: 0.2 m; verbatimLatitude: 25º34’55’’N; verbatimLongitude: 109º6’53’’W; **Identification:** identifiedBy: María Ana Tovar-Hernández; **Event:** samplingEffort: 2 collectors, 2 hours sampling; year: 2021; month: April; day: 8; habitat: Estuary; fieldNumber: TOP-S-20210408-2; **Record Level:** institutionID: ECOSUR; collectionID: ECOSUR QNR.IN.021.0497; collectionCode: ECOSUR 0306
- **Type status:** Paratype. **Occurrence:** recordNumber: TOP-S-20210408-2; individualCount: 4; occurrenceID: 365BD068-520D-53AD-8867-6E482612AD6E; **Taxon:** kingdom: Animalia; phylum: Annelida; class: Polychaeta; order: Terebellomorpha; family: Pectinariidae; genus: Pectinaria; specificEpithet: *santii*; taxonRank: species; taxonomicStatus: a; **Location:** higherGeography: North America, México, Gulf of California; continent: America; waterBody: Gulf of California; country: Mexico; countryCode: MX; stateProvince: Sinaloa; municipality: Ahome; locality: El Mavirí; verbatimDepth: 0.2 m; verbatimLatitude: 25º34’55’’N; verbatimLongitude: 109º6’53’’W; **Identification:** identifiedBy: María Ana Tovar-Hernández; **Event:** samplingEffort: 2 collectors, 2 hours sampling; year: 2021; month: April; day: 8; habitat: Estuary; fieldNumber: TOP-S-20210408-2; **Record Level:** institutionID: GEOMARE; collectionID: GEOMARE; collectionCode: GEOMARE 009
- **Type status:** Other material. **Occurrence:** recordNumber: MANA-S-20210520; recordedBy: Patricia Salazar-Silva; individualCount: 2; occurrenceID: CF9988FC-092B-594B-AC5F-543BB619E34E; **Taxon:** kingdom: Animalia; phylum: Annelida; class: Polychaeta; order: Terebellomorpha; family: Pectinariidae; genus: Pectinaria; specificEpithet: *santii*; taxonRank: species; **Location:** higherGeography: North America, México, Nayarit; continent: America; country: Mexico; countryCode: MX; stateProvince: Nayarit; municipality: Santiago Ixcuintla; locality: Boca del Camichín; verbatimDepth: 0.2 m; verbatimLatitude: 21°44'38.0" N; verbatimLongitude: 105°29'26.2" W; **Identification:** identifiedBy: María Ana Tovar-Hernández; **Event:** year: 2021; month: May; day: 20; habitat: Estuary; fieldNumber: MANA-S-20210520; **Record Level:** institutionID: UNAM-ICML; collectionID: ICML-EMU; collectionCode: ICML-EMU 13454

#### Description

**Figure 2.**
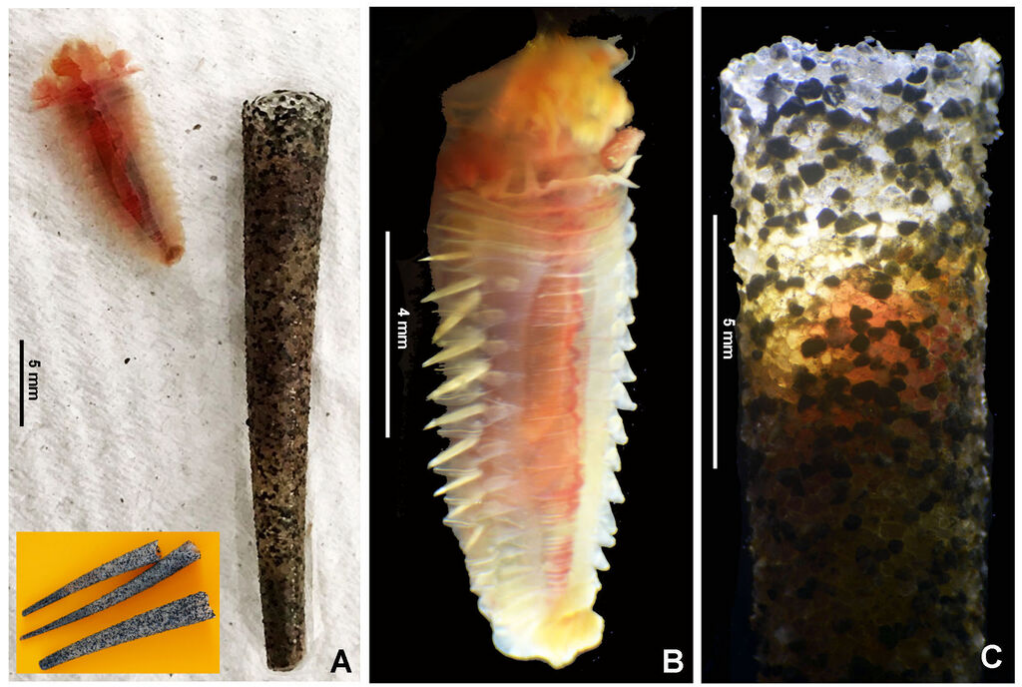
Live *Pectinariasantii* sp. n. (holotype SCRIPPS SIO-BIC A13452). **A** Worm and tubes; **B** worm, ventral view; **C** anterior end of tube.

**Figure 3.**
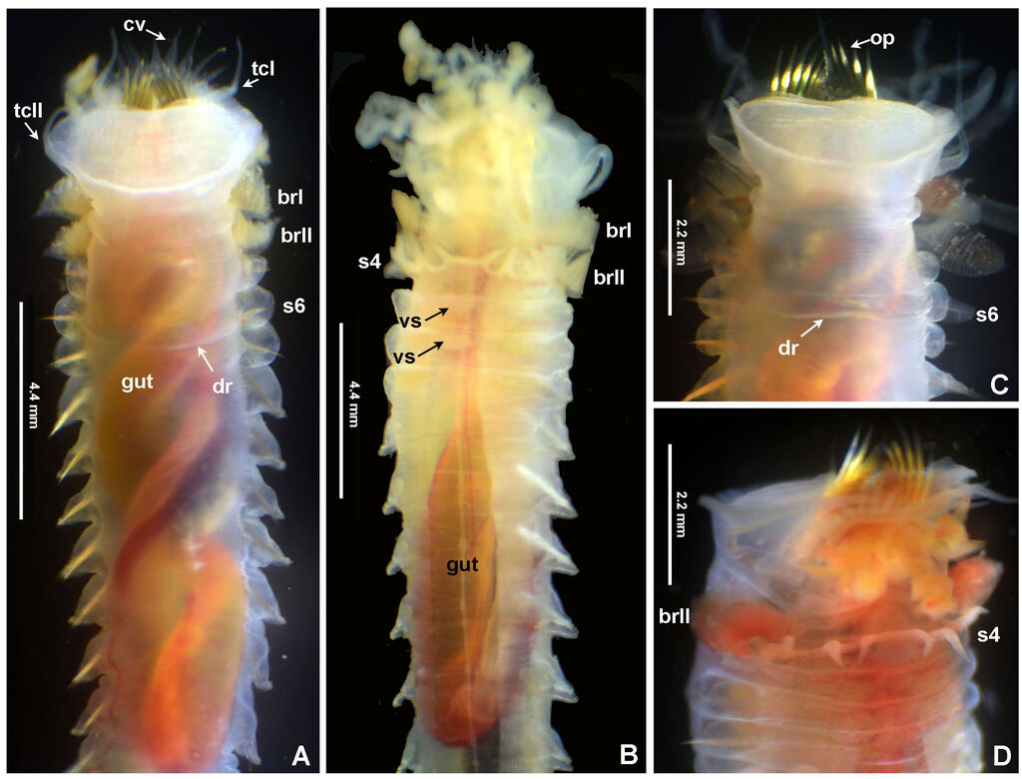
Alive *Pectinariasantii* sp. n. (holotype SCRIPPS SIO-BIC A13452). **A**, **C** Anterior end, dorsal views; **B**-**D** same, ventral views. Abbreviations: brI = branchia 1, brII = branchia 2, cv = cephalic veil, dr = dorsal ridge, op = opercular paleae, s = segment, s4 = segment 4, s6 = segment 6, tcI = tentacular cirrus 1, tcII = tentacular cirrus 2, vs = ventral glandular shield.

**Figure 4.**
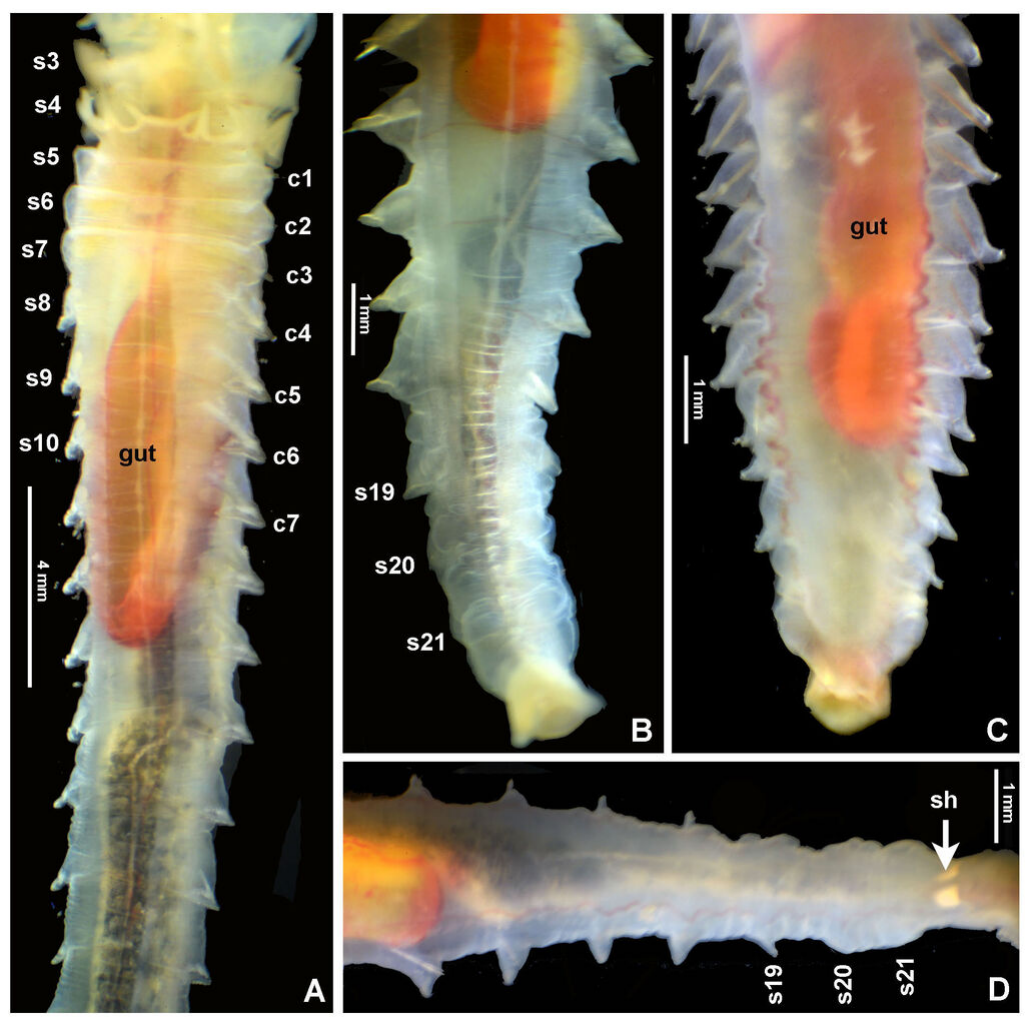
Alive *Pectinariasantii* sp. n. (holotype SCRIPPS SIO-BIC A13452). **A** Body trunk, ventral view; **B**-**D** posterior ends, dorsal view. Abbreviations: sh = scaphal hooks. Numbers refer to segments (s) or chaetigers (c).

**Figure 5.**
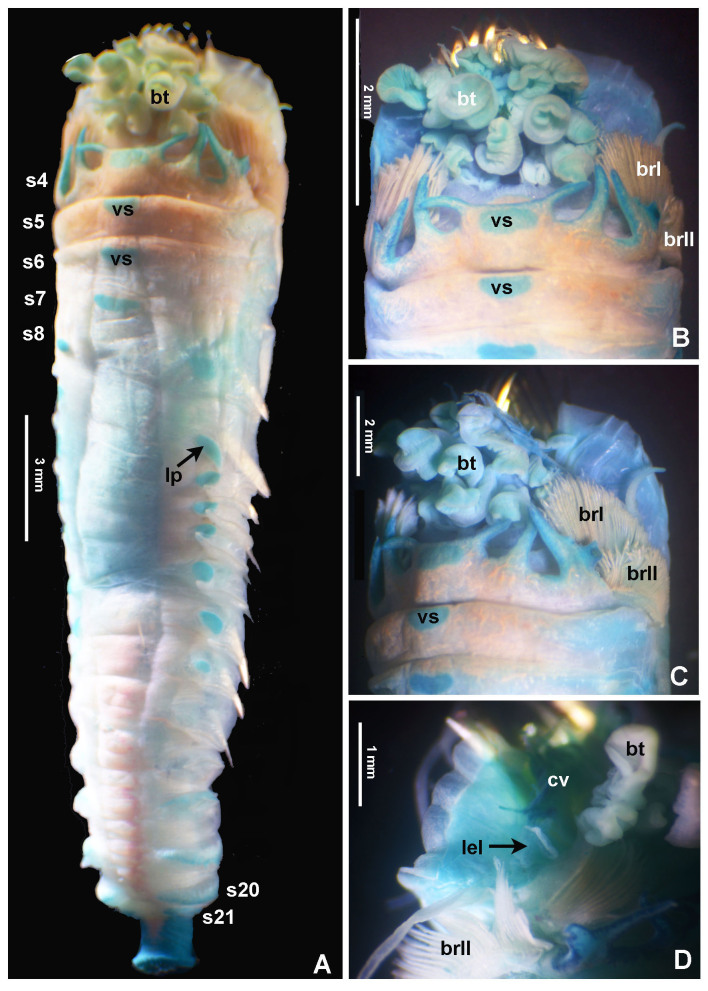
Selected features of *Pectinariasantii* sp. n., stained with methyl green (holotype SCRIPPS SIO-BIC A13452). **A** Body, ventral view; **B**-**D** anterior end, ventral and ventro-lateral views. Abbreviations: bt = buccal tentacle, brI = branchia 1, brII = branchia 2, cv = cephalic veil, lp = lateral pads, vs = ventral shield. Numbers refer to segments (s) or chaetigers (c).

**Figure 6.**
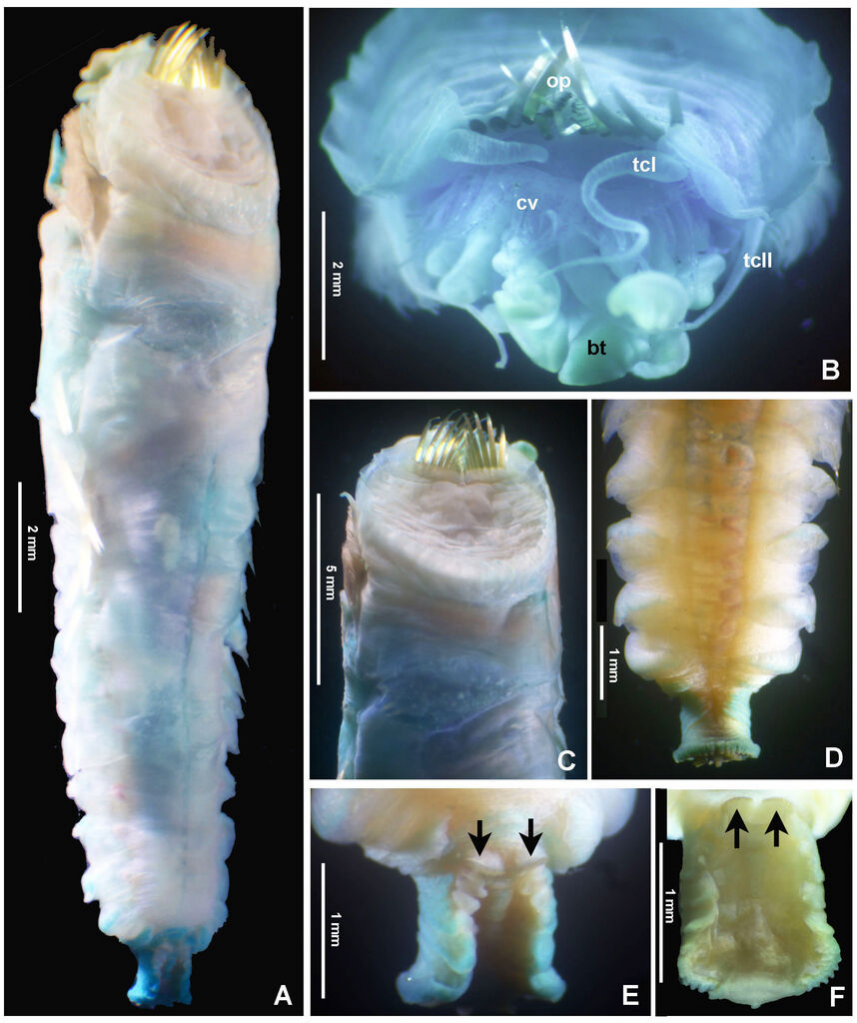
Selected features of *Pectinariasantii* sp. n., stained with methyl green (**A, C**: paratype 4 UANL 8152; **B**: paratype 3 UANL 8152; **D–E**: paratype 14 CNAP-ICML UNAM POP-67-001; **F**: paratype 21 ECOSUR 0306). **A** Body, dorso-lateral view; **B** cephalic area; **C** operculum; **D**-**F** scaphe, ventral and dorsal views. Arrows in E and F point to scaphal hooks. Abbreviations: bt = buccal tentacle, cv = cephalic veil, lel = lateral ear-shaped lobe, op = opercular paleae, tcI = tentacular cirrus 1, tcII = tentacular cirrus 2.

**Figure 7.**
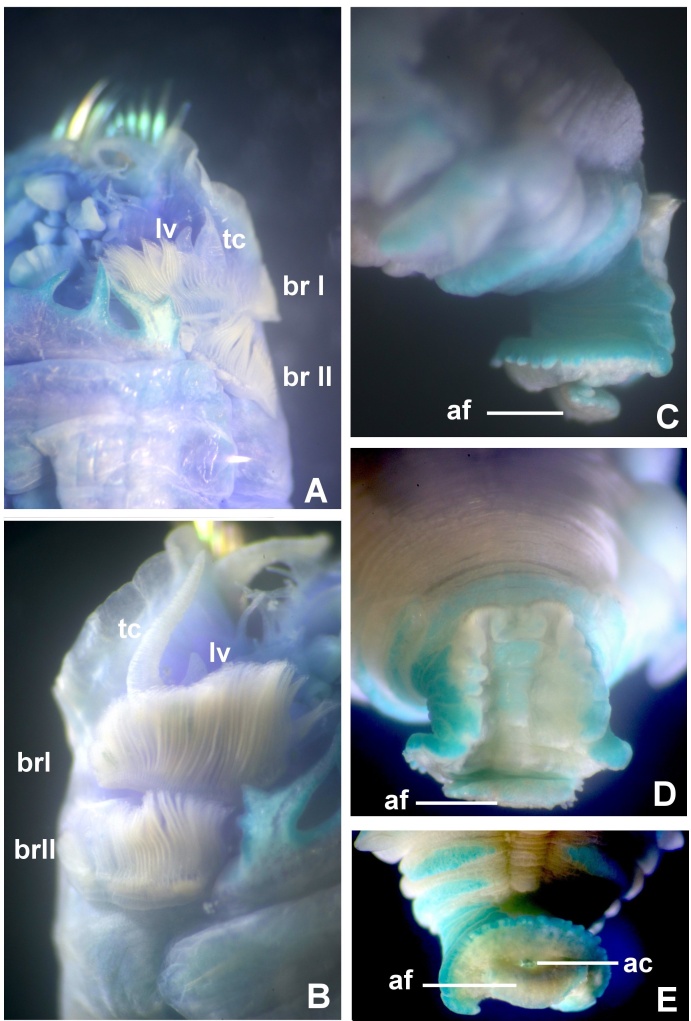
Morphological features and details of notochaetae in *Pectinariasantii* sp. n. (paratype 6 UANL 8152) **A-B** Branchiae, tentacular cirrus and ventral lobe in S1; **C-E** Scaphe and anal flap, lateral, dorsal and apical views, respectively. Abbreviations: ac = anal cirrus, af = anal flap, brI = branchia 1, brII = branchia 2, lv = ventral lobe in S1, tc = tentacular cirrus.

**Figure 8.**
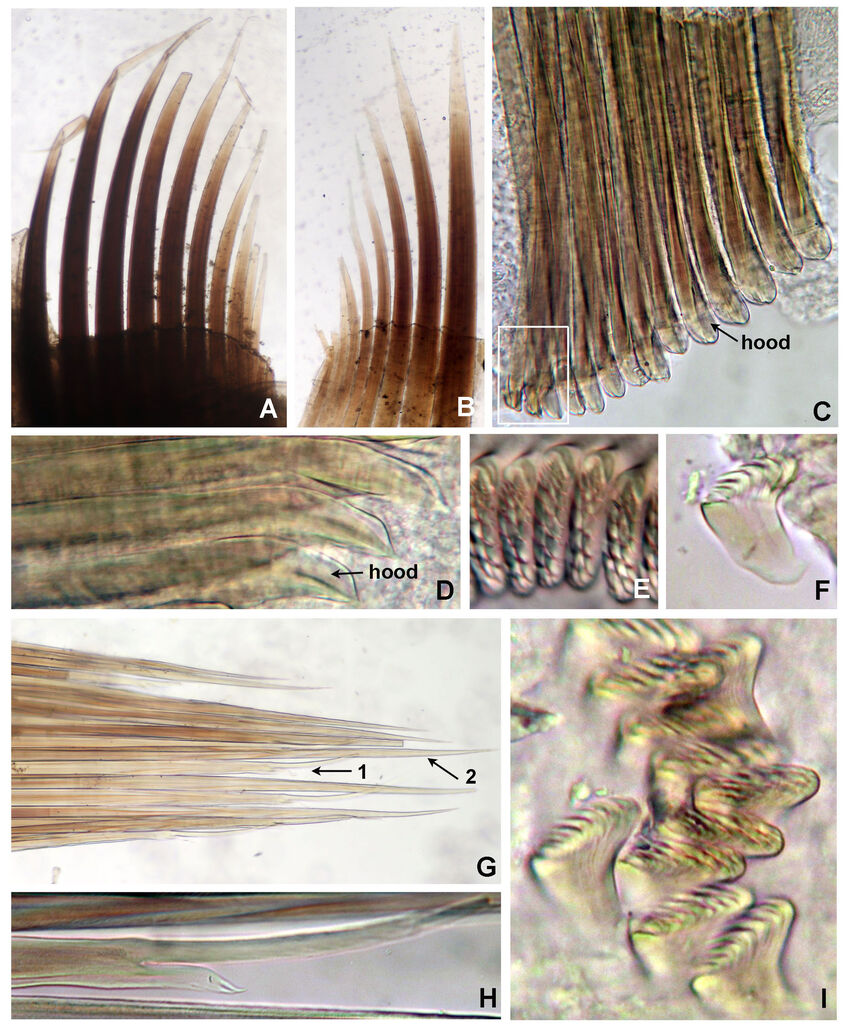
Chaetae and uncini of *Pectinariasantii* sp. n. (paratype 11 ICML-EMU 13290). **A, B** Opercular paleae; **C** entire row of hooded scaphal hooks, white rectangle shows the innermost hooks; **D** innermost scaphal hooks; **E** uncini, front-lateral view; **F** uncinus, lateral view; **G** notochaetae: 1 chaeta from anterior row, 2 chaeta from posterior row; **H** chaeta from anterior row with a mid-incision, bifid antero-lateral tip and smooth margins; **I** uncini, different views. A–B) 40x, C, G) 100x, D–F, H) 400x, I) 1000x magnification.

**Figure 9.**
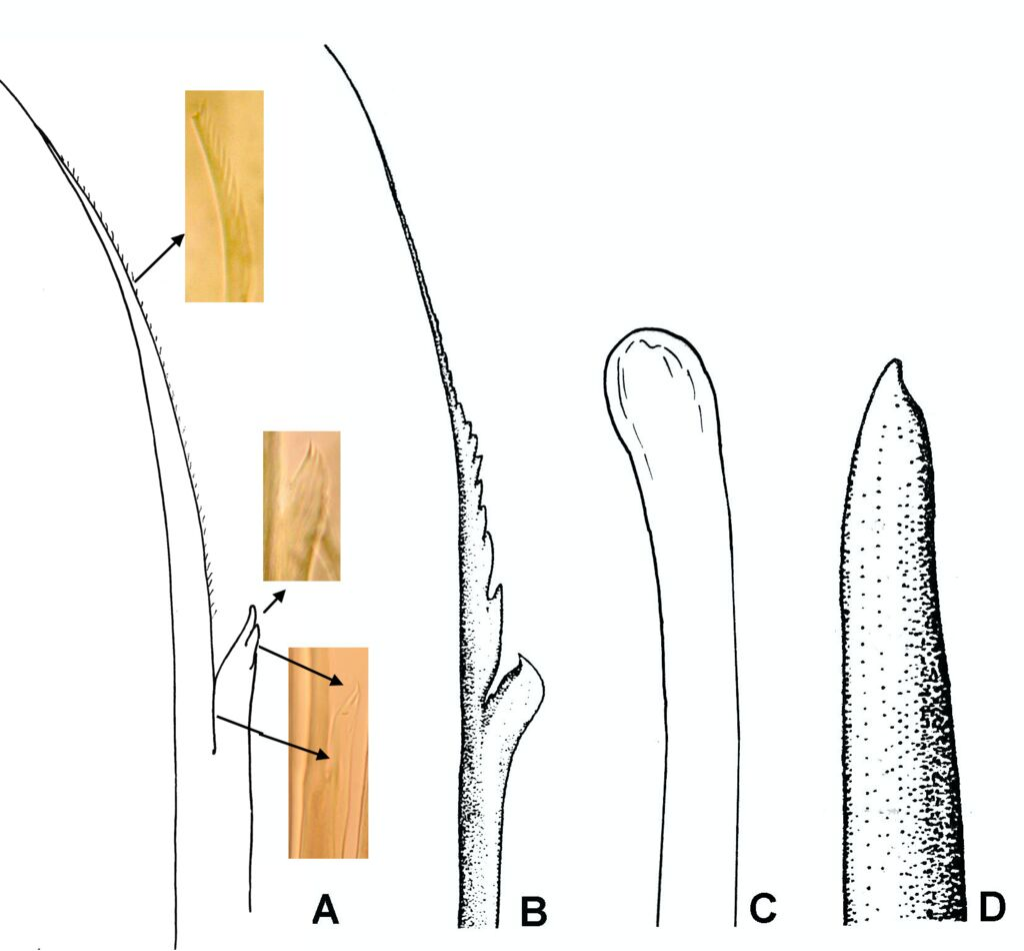
Notochaetae from anterior row and scaphal hooks in *P.santii* sp. n. and *P.hartmanae*. **A, C** *Pectinariasantii* sp. n., chaeta (A) and hook (C); **B, D** *P.hartmanae*, modified from Reish, 1968, chaeta (B) and hook (D).

Typical ice cream cone shaped tubes (Fig. 2A and C), composed of a single layer of cemented sand grains, translucent and black (Fig. 2A and C). Conical body, stout (Fig. 2A–B), 22.6 mm long including scaphe (8.3–26 mm) and 4.2 mm width at chaetiger 3 (2.2–5.4 mm). Preserved types pale cream with dorsum mostly translucent on its entire length. Operculum 3.5 mm width (2.3–5.3 mm) with margin raised with smooth edge (Fig. 3A and C, Fig. 6A and C) and two bundles of paleae (Fig. 3A and C–D), each bundle with 9 (8–10) stout, flattened golden palaea (Fig. 3A and C–D) curved and tapering to a pointed tip (Fig. 8A–B). Cephalic veil free from operculum, distal margin with 20 (18–25) slender cirri of variable sizes (Fig. 6B). A pair of ear-shaped lobes adjacent to both sides of dorso-lateral base of cephalic veil (Fig. 5D). Few blunt buccal tentacles (short or long depending on contraction after relaxation or fixation) distributed along the rim (Fig. 5A–C and Fig. 6B). Segment 1 with a pair of long tentacular cirri arising from antero-ventral edge near outer most paleae, thin, elongate distally pointed (Fig. 3A and Fig. 6B); and a pair of small ventral lobes (Fig. 7A–B). Segment 2 with a pair of tentacular cirri, similar to those present on segment 1, but shorter and located laterally (Fig. 3A and Fig. 6B). Posterodorsal lobe on segment 2 absent. Two pairs of pectinated stalked branchiae, on segments 3 and 4 (Fig. 3A–D, Fig. 5B–D and Fig. 7A–B), each pair consisting of numerous loose, flat and smooth lamellae, much higher than broad (Fig. 5B–C). Branchiae from segment 3 inserted ventro-laterally, longer than those in segment 4, which is inserted laterally (Fig. 5C and Fig. 7A–B). Segment 4 with a ventral crest with six anterior projections: two median with horn-shaped tips directed outwards; two central with horns directed inwards and two lateral directed inwards, but with a broad base and a small external knob (Fig. 3B, D, Fig. 4A and Fig. 5A–C). Segments 4, 5, 6 and 7 with ventral glandular pads becoming progressively more lateral and broader on segments 6 and 7. In mid-ventral area of each glandular pad, there is a small ventral shield blue when stained with methyl green (Fig. 5A–B). Segment 6 with a dorsal ridge, whitish in live specimens (Fig. 3A and C). Lateral glandular pads on segments 8 to 15 (8 pads), diminishing gradually in size towards posterior segments (Fig. 5A). Notopodia beginning on segment 5 (Fig. 4A), extending until segment 19 (3 chaetigers with notopodia only: S5, 6 and 7) (Fig. 5A); neuropodia beginning on segment 8, extending until segment 19 (12 chaetigers with noto- and neuropodia); segments 20 and 21 achaetous (Fig. 4B and Fig. 5A). Notopodia with two kinds of chaetae forming a bundle (Fig. 8G): chaetae from anterior row with deep incision resembling a shaft and blade (Fig. 8H and Fig. 9A), where internal basal margin of blade is smooth, then covered progressively with tiny serrations from mid-anterior portion to tip (Fig. 9A) and an apparently bifid process at the lateral end of shaft (Fig. 8G_1,_ H and Fig. 9A) or with narrow and delicate serrations (Fig. 9A); chaetae from posterior row long, apparently narrowly limbate (Fig. 8G_2_). Neurochaeta as pectinate uncini (Fig. 8I), major teeth surmounted by three rows of small teeth, 7 teeth per row (Fig. 8E); anterior peg nearly U-shaped; stout handle directed posteriorly, as long as the distance between front and back (Fig. 8F). Scaphe distinctly separated from segment 21, formed by fusion of five posterior segments (Fig. 4B–C, Fig. 5A, Fig. 6A and D–F). Fifteen pairs of scaphal hooks (13–21 in paratypes) located at dorsolateral region of scaphe (Fig. 4D and Fig. 6E–F), amber coloured, long, with distal margin hooded nearly rounded and slightly curved in most hooks (Fig. 8C and Fig. 9C), except for 3–4 innermost hooks with attenuated and slightly curved distal margin (Fig. 8D). Scaphe funnel-shaped (Fig. 6A), rounded ventrally (Fig. 6D), concave dorsally forming a groove (Fig. 6E–F and Fig. 7B), with six lateral crenulations (Fig. 6F and Fig. 7B). Anal flap is round-leaf shaped (Fig. 7C–E) with reminiscent crenulations bordering its margin and a small, rounded mid-dorsal anal cirrus (Fig. 6F and Fig. 7C–E).

Colour in live specimens: tegument entirely translucent in live worms (Figs 3, 4), with the opercular margin whitish (Fig. 3A and C) and small nearly rectangular ventral glandular pads or shields on segments 4–7, also whitish (Fig. 3B). Branchiae, blood vessels and some areas of gut red coloured (Fig. 3A–D and Fig. 4A).

##### Variation

**Figure 10.**
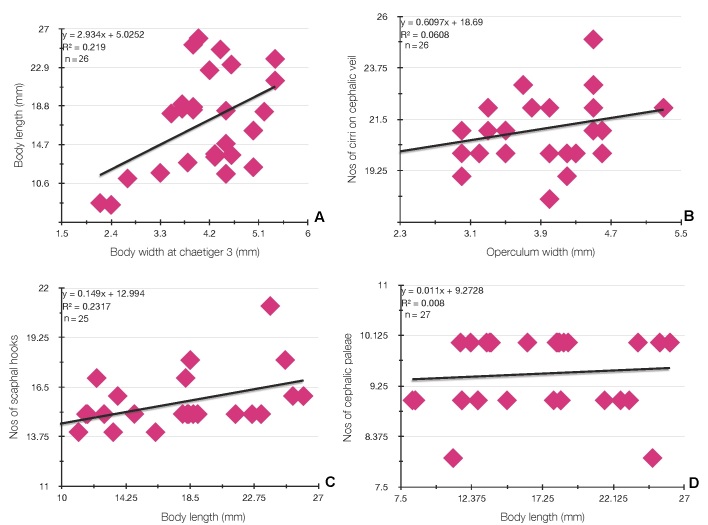
Relationships between biometrical features in *Pectinariasantii* sp. n. **A** Body length and body width; **B** number of cirri on cephalic veil and operculum width; **C** number of scaphal hooks and body length; **D** number of cephalic paleae and body length.

At least two features are stable amongst the series of types: the number of chaetigers with notopodia (from S5–19 and 3 chaetigers with notopodia only: S5, 6 and 7) and those with noto and neuropodia (12 from S8–19) and the shape of ventral crest on segment 4, with tips horn-shaped. Body length was significantly correlated with body width (Fig. 10A), but the number of cirri on the cephalic veil (Fig. 10B), scaphal hooks (Fig. 10C) and cephalic paleae (Fig. 10D) does not significantly vary with operculum width and body length.

#### Diagnosis

Segment 4 with a ventral crest with six horn-shaped anterior projections; three chaetigers with notopodia (from S5, S6 and S7); 12 chaetigers with noto- and neuropodia (from S8–19); 8–10 golden opercular paleae per lobe; a pair of ear-shaped lobes at base of cephalic veil; anterior row of chaetae in notopodia with a deep incision and an apparently bifid process at the lateral end of shaft; and 13–21 pairs of amber scaphal hooks with distal margin rounded and hooded.

#### Etymology

Named after the first author's son Santiago Hernández celebrating his 11th birthday. He also patiently helped us collect the magnificent ice cream cone worms and enjoyed seeing these worms live under the microscopy as much as we did.

#### Taxon discussion

A comparative table of the major diagnostic characters of some *Pectinaria* species was provided by Zhang and Qiu (2017) and Zhang et al. (2022). *Pectinariagouldii* (Verrill, 1874) (Verrill 1874), *P.hartmanae* Reish, 1968 (Reish 1968), *P.nana* Wesenberg-Lund, 1949 (Wesenberg-Lund 1949), *P.longispinnis* Grube, 1878 (Grube 1878) and *Pectinariasantii* sp. n. share the presence of notochaetae on segments 5–19 and neurochaetae on segments 8–19. However, *P.gouldii*, *P.nana* and *P.longispinnis* have pointed scaphal hooks (straight or strongly curved); hooks in *P.hartmanae* are blunt (with shaft decreasing gradually towards the tip) and, in *P.santii* sp. n., hooks are rounded (shafts increasing gradually in width towards the tip, forming rounded tips in most hooks, except in the innermost, which are oval with pointed tips).

Moreover, there are other four differences in *P.hartmanae* Reish, 1968 (Reish 1968) and the new species from central-eastern Gulf of California: 1) a ventral crest on segment 4 with distinctive horns was not described in *P.hartmanae* by Reish and their absence was corroborated by Dr. Karen Osborn in holotype and paratypes (pers. comm, July 2021); 2) Reish (1968) reported the presence of 8 to 10 pairs of scaphal hooks in *P.hartmanae*, whereas *Pectinariasantii* sp. n. have a high number (13–21 pairs); 3) The blades of anterior row of notochaetae in *P.hartmanae* have a coarsely basal denticulate margin (Fig. 9B), whereas it is smooth, then covered progressively with tiny serrations from mid-anterior portion to tip in *Pectinariasantii* sp. n. (Fig. 9A and Fig. 7G); an entire, nearly rounded lateral end of shaft in *P.hartmanae* (Fig. 9B), but it is bifid apparently in *Pectinariasantii* sp. n. (Fig. 8H and Fig. 9A) or with narrow and delicate serrations (Fig. 10H); and 4) uncini in *P.hartmanae* were described as having two rows of major teeth, whereas there are three rows of uncini in *Pectinariasantii* sp. n. However, as serrations of notochaeta are difficult to see under light microscopy (100x) (Fig. 10F-H), it is desirable to examine these carefully with scanning electron microscopy.

The ventral crest on segment 4 with distinctive horns, constitutes a stable character in all types examined of *P.santii* sp. n. A similar feature was described recently in *Pectinarializhei* Zhang, Hutchings & Qiu, 2022 (Zhang et al. 2022), but it has small crenulations in the ventral-most part, whereas the ventral-most part is smooth in *P.santii* sp. n. In addition, *P.lizhei* have notochaetae on S5–S20 and neurochaetae on S8–S20 (notochaeta on S5–S7 and neurochaeta on S8–S19 in *P.santii* sp. n). A ventral crenulate margin in segment 6 was reported for *P.antipoda* Schmarda, 1861 (Schmarda 1861) (Zhang and Hutchings 2019), but it lacks the horns present in *P.santii* sp. n. and *P.lizhei*.

Compared to other *Pectinaria* species from the Temperate Northern Pacific, *P.santii* sp. n. has 12 biramous chaetigers (in segments 8–19), whereas there are 13 biramous chaetigers in *P.californiensis* and *P.newportensis* (segments 8–20). There are also differences in the number of cephalic paleae (not reported in *P.californiensis*, 9–13 in *P.newportensis*, 8–10 in *P.santii* sp. n.) and number of cirri on the cephalic veil (18–30 in *P.californiensis*, 19–24 in *P.newportensis*, 18–25 in *P.santii* sp. n.). Additionally, *P.santii* sp. n. differs from *P.californiensis* and *P.newportensis* by having golden opercular paleae (copper in colour in *P.californiensis* and *P.newportensis*). However, it is highly recommended to examine the types of both Californian species to properly describe and illustrate features that were omitted in original descriptions, which is out the scope of the present contribution. Hartman’s types are housed at the Natural History Museum of Los Angeles County (AHF 34 and 35 to *P.californiensis* and *P.newportensis*, respectively).

## Identification Keys

### Taxonomic key to species of *Pectinaria* from the Tropical Eastern Pacific and Temperate Northern Pacific

**Table:** 

1	Scaphal hooks straight, with distal margins rounded or blunt	2
–	Scaphal hooks curved, hooked distally	3
2	Segment 4 with six anterior horn-like projections on ventral side	*P.santii* sp. n.
–	Segment 4 without any projections on ventral side	*P.hartmanae* Reish, 1968
3	Scaphal hooks gently curved distally; opercular paleae copper in colour	*P.californiensis* Hartman, 1941
–	Scaphal hooks strongly curved distally; opercular paleae yellow	*P.newportensis* Hartman, 1941

## Supplementary Material

XML Treatment for Pectinaria santii

## Figures and Tables

**Table 1.** Major quantitative morphological parameters in *Pectinariasantii* sp. n.

Specimen	Body length (mm)	Body width at chaetiger 3 (mm)	Number of cirri on cephalic veil	Number of paleae (right bundle)	Operculum width (mm)	Number of scaphal hooks (right bundle)
Holotype SIO-BIC A13452	22.6	4.2	20	9	3.5	15
Paratype 1 UANL 8152	18.4	3.9	21	10	3	15
Paratype 2 UANL 8152	18	3.5	19	9	3	15
Paratype 3 UANL 8152	-	3.7	22	10	4	-
Paratype 4 UANL 8152	21.5	5.4	20	9	4.6	15
Paratype 5 UANL 8152	24.8	4.4	22	8	4.5	18
Paratype 6 UANL 8152	18.7	3.9	22	10	4	15
Paratype 7 UANL 8152	18.3	4.5	23	10	4.5	15
Paratype 8 ICML-EMU-13290	13.4	4.3	21	10	3.5	14
Paratype 9 ICML-EMU-13290	18.2	5.2	25	10	4.5	17
Paratype 10 ICML-EMU-13290	14.8	4.5	23	9	3.7	15
Paratype 11 ICML-EMU-13290	26	4	20	10	4.2	16
Paratype 12 ICML-EMU-13290	16.2	5	19	10	4.2	14
Paratype 13 CNAP-ICML UNAM POP-67-001	18.5	3.7	22	9	3.3	18
Paratype 14 CNAP-ICML UNAM POP-67-001	23.8	5.4	22	10	5.3	21
Paratype 15 CNAP-ICML UNAM POP-67-001	11.1	2.7	21	8	4.6	14
Paratype 16 CNAP-ICML UNAM POP-67-001	23.2	4.6	20	9	4	15
Paratype 17 CNAP-ICML UNAM POP-67-001	8.3	2.4	22	9	3.3	15
Paratype 18 ECOSUR 0306	12.3	5	18	10	4	17
Paratype 19 GEOMARE 009	13.6	4.6	21	10	3.3	-
Paratype 20 ECOSUR 0306	12.8	3.8	22	9	3.8	15
Paratype 21 ECOSUR 0306	11.7	3.3	20	9	3	15
Paratype 22 ECOSUR 0306	25.3	3.9	21	10	4.5	16
Paratype 23 GEOMARE 009	11.6	4.5	20	10	4.3	15
Paratype 24 GEOMARE 009	19	3.7	20	10	3.2	15
Paratype 25 GEOMARE 009	13.7	4.3	22	10	4	16
Paratype 26 ECOSUR 0306	8.5	2.2	-	9	2.3	13
